# Supplementary material for: Decision-making in plants under competition
Source: Nat Commun. 2017 Dec 21;8:2235. doi: 10.1038/s41467-017-02147-2 (PMC5740169; doi:10.1038/s41467-017-02147-2)
Supplement: Supplementary file 1 — Supplementary Information [file 41467_2017_2147_MOESM1_ESM.pdf]

Supplementary Figure 1

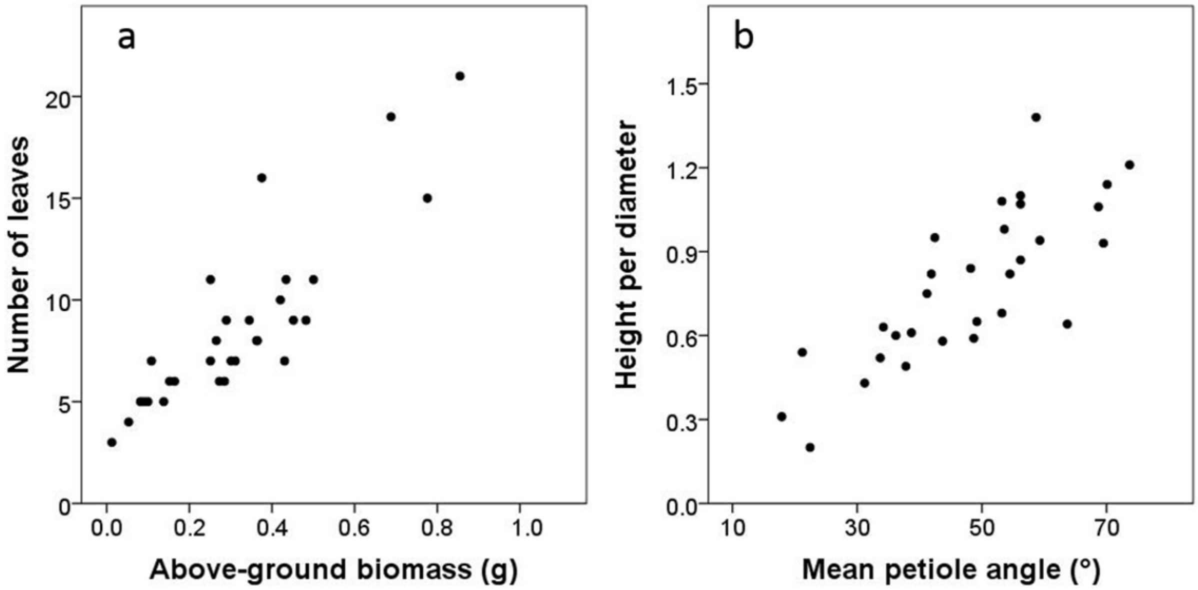

Correlations between leaf number and ramet biomass (a) and between height-per-diameter ratio and mean petiole angle (b) in *Potentilla reptans*. Results for Pearson correlation in a: $r = 0.872$, $P < 0.001$, $n = 30$; and b: $r = 0.804$, $P < 0.001$, $n = 30$.
