# Supplementary material for: Decision-making in plants under competition
Source: Nat Commun. 2017 Dec 21;8:2235. doi: 10.1038/s41467-017-02147-2 (PMC5740169; doi:10.1038/s41467-017-02147-2)
Supplement: Supplementary file 3 — Supplementary Information [file 41467_2017_2147_MOESM3_ESM.pdf]

**Description of Additional Supplementary Files**

**File Name: Supplementary Data 1**

Description: Source data for the manuscript, namely the measured responses of *Potentilla reptans* to the different treatments, including number of leaves, number of newly-produced leaves, petiole length, plant height, plant diameter, height per diameter, leaf area, specific leaf area (SLA), total stolon length and mean internode length.
